# Supplementary material for: Lysosomal Network Defects in Early-Onset Parkinson’s Disease Patients Carrying Rare Variants in Lysosomal Hydrolytic Enzyme Genes
Source: Int J Mol Sci. 2025 Sep 27;26(19):9454. doi: 10.3390/ijms26199454 (PMC12525278; doi:10.3390/ijms26199454)
Supplement: Supplementary file 1 [file ijms-26-09454-s001.zip › ijms-3869459-supplementary.pdf]

Lysosomal network defects in early-onset Parkinson's disease patients carrying rare variants in lysosomal hydrolytic enzyme genes

Alba Pascual, Thaleia Moulka, Oriol de Fàbregues, Roberta Repossi, Pedro J García-Ruiz, Saida Ortolano, Marisel De Lucca, Lydia Vela-Desojo, Marta Alves-Villar, Marcos Frías, Cici Feliz-Feliz, Mònica Roldán, Jonathan Olival, Guerau Fernàndez, Francesc Palau, Jordi Pijuan and Janet Hoenicka

SUPPLEMENTARY DATA - TABLE OF CONTENTS

SUPPLEMENTARY FIGURES

Supplementary Figure 1. Clinical brain testing in PD-302 patient. page 3
Supplementary Figure 2. Gb3 levels are not altered in PD-302 fibroblasts. page 4

SUPPLEMENTARY TABLES

Supplementary Table 1. Knowledge-driven Analysis criteria, tools, and scores applied for variant prioritization in gene candidate selection. page 5

Supplementary Table 2. Detailed genetic information, pathways, and KDA scores of genetic variants prioritized in patient PD-302. page 6

Supplementary Table 3. Detailed genetic information, pathways, and KDA scores of genetic variants prioritized in patient PD-227. page 7

Supplementary Table 4. Detailed genetic information, pathways, and KDA scores of genetic variants prioritized in patient PD-216. page 8

Supplementary Table 5. Summary of *GLA* variants in the EOPD literature: reported cohorts and case studies. pages 9-10

Supplementary Table 6. *In silico* predicted effects of *GLA* (p.Asp313Tyr) and *GLB1* (p.Arg419Gln) variants found in the patients PD-302, PD-227, and PD-216. page 11

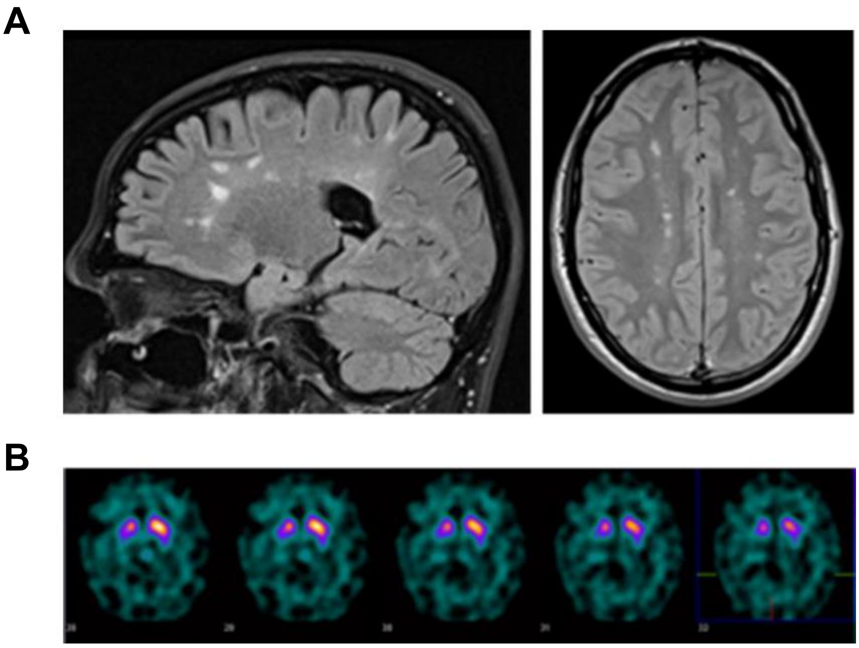

Supplementary Figure 1. Clinical brain testing in the PD-302 patient. A) Magnetic Resonance Imaging (MRI) showed multiple demyelinating lesions. **B**) DaTScan: Dopamine transporter (DAT) single-photon emission computed tomography demonstrated the loss of striatal DAT.

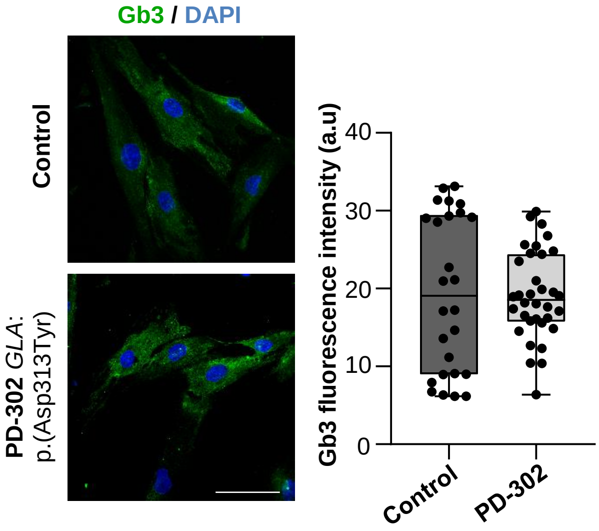

Supplementary Figure 2. Gb3 levels are not altered in the PD-302 fibroblasts. Representative immunofluorescence images of globotriaosylceramide (Gb3, green) and nuclei (DAPI, blue) in fibroblasts from the patient (PD-302, *GLA*:p.(Asp313Tyr)) and control. Quantification of Gb3 fluorescence intensity is shown as a box plot (25th percentile, median, 75th percentile; whiskers represent minimum and maximum values; n = 3 independent biological replicates). Mann–Whitney *U*-test. Scale bars: 50 µm. Abbreviation: a.u, arbitrary units.

Supplementary Table 1. Criteria, tools, and scores for knowledge-driven analysis used in gene candidate prioritization.

Tool	Description	Prediction	Score
Pathogenicity predictors			
**CADD**	A tool for scoring the deleteriousness of single-nucleotide variants, multi-nucleotide substitutions, as well as insertion/deletion variants in the human genome.	≥ 30	2
		15-29.9	1
**SIFT**	Predicts whether an amino acid substitution affects protein function based on sequence homology and the physical properties of amino acids.	Intolerant	1
		Tolerant	0
**MutationTaster2**	Evaluates DNA sequence variants to determine their potential to cause diseases.	Disease-causing	1
		Benign	0
**PolyPhen2**	Predicts possible impact of an amino acid substitution on the structure and function of a human protein using straightforward physical and comparative considerations.	Possibly/Probably damaging	1
		Benign	0
**FATHMM-MKL**	Predicts the functional consequences of non-coding and coding SNVs.	Damaging/Uncertain	1
		Neutral	0
**SpliceAI**	A deep learning-based tool for identifying splicing variants.	Splicing alteration	1
		No alteration	0
Conservation predictor			
**GERP++**	Identify constrained elements in multiple alignments by quantifying substitution deficits. These deficits represent substitutions that would have occurred if the element were neutral DNA, but did not occur because the element has been under functional constraint.	≥2	1
		<2	0
Variant rarity			
**gnomAD**	Resource to aggregate and harmonize both exome and genome sequencing data from a wide variety of large-scale sequencing projects. Minor Allele Frequency (MAF) from European non-Finnish population.	Variant Not Found (NF)	3
		MAF < 0.001	2
		0.01 > MAF > 0.001	1
ACMG variant classification			
**Franklin**	A web-based tool that relies on ACMG guidelines to classify the pathogenicity of clinically observed variants.	Pathogenic / Likely Pathogenic	3
		Variant of Uncertain Significance (VUS)	2
		Likely Benign / Benign	1
Gene constraint			
**gnomAD**	Variant tolerant or intolerant gene. Z-score value for missense variants, pLI score for loss of function variants.	Z-score > 3.09 or pLI > 0.5	1
		Z-score < 3.09 or pLI < 0.5	0
Gene expression in targeted organ/tissue			
**Human Protein Atlas**	Program to map all the human proteins in cells, tissues, and organs using an integration of various omics technologies, including antibody-based imaging, mass spectrometry-based proteomics, transcriptomics, and systems biology.	Brain specific expression or low human brain regional specificity, but there is	1

Supplementary Table 2. Detailed genetic information, pathways, and KDA scores of genetic variants prioritized in patient PD-302.

General Information				Predictor Scores														
Gene (Inheritance *)	Pathway	Variant	INH	ACMG	CADD	Freq	ACMG	CADD	Freq	MT	PolP2	SIFT	F-MKL	S-AI	GERP	Z/pLI	BE	TS
FBXO7 (AR)	LY/ATP	p.(Ala248Thr)/c.742G>A	M	VUS	24.5	3.97E-06	2	1	3	1	1	1	1	0	1	0	1	12
MMAB (AR)	COB	p.(His183Leu)/c.548A>T	F	P	23.7	8.83E-06	3	1	2	1	1	1	1	0	1	0	1	12
SLC25A5	LY/ATP/VT/NR	p.(Phe82Ile)/c.244T>A	F	VUS	25.5	8.94E-04	2	1	2	1	1	1	1	0	1	0	1	11
USP13	LY/ATP	p.(Pro631Thr)/c.1891C>A	F	VUS	26.8	4.19E-04	2	1	2	1	1	1	1	0	1	0	1	11
LRRK2 (AD)	LY/VT/NR	p.(Phe1436Leu)/c.4306T>C	M	VUS	24.1	NF	2	1	3	1	1	0	1	0	1	0	1	11
CUX1 (AD)	VT	p.(Val1040Met)/c.3118G>A	M	VUS	19.6	1.08E-04	2	1	2	1	1	0	1	0	1	1	1	11
ABCA12 (AR)	VT	p.(Phe67Tyr)/c.200T>A	M	VUS	25.6	NF	2	1	3	0	1	1	1	0	1	0	1	11
EXTL3 (AR)	GB	p.(Val463Met)/c.1387G>A	F	VUS	21.3	NF	2	1	3	1	0	1	1	0	1	0	1	11
SPTA1 (AR)	LY/ATP/VT/NR	p.Glu1115*/c.3343G>T	F	LP	40.0	1.20E-04	3	2	3	1	NA	NA	.	0	1	0	0	10
GFAP (AD)	LY/ATP	p.(Gln382Glu)/c.1144C>G	F	VUS	25.8	1.87E-05	2	1	2	1	1	0	1	0	1	0	1	10
ADORA1	NR	p.(Thr75Ile)/c.224C>T	M	VUS	22.2	NF	2	1	3	1	0	0	1	0	1	0	1	10
ATXN1 (AD)	NR	p.(Gly258Cys)/c.772G>T	M	VUS	24.5	2.83E-03	2	1	1	1	1	1	1	0	1	0	1	10
GORASP1	VT	p.(Asp317Tyr)/c.949G>T	F	B	28.4	8.95E-03	1	1	1	1	1	1	1	1	1	0	1	10
BICD2 (AD)	VT	p.(Thr649Met)/c.1946C>T	F	VUS	28.0	1.55E-05	2	1	2	1	1	0	1	0	1	0	1	10
ACHE	LY/ATP/NR	p.(Gly89Glu)/c.266G>A	F	B	24.1	6.25E-04	1	1	2	1	1	0	1	0	1	0	1	9
SPTBN4 (AR)	LY/ATP/VT/NR	p.(Glu1193Lys)/c.3577G>A	M	LB	24.9	1.50E-03	1	1	1	1	1	0	1	0	1	1	1	9
NFE2L2 (AD)	GLG/MIT/ER/NR	p.(Leu309Phe)/c.925C>T	F	B	24.2	2.91E-03	1	1	1	1	1	1	1	0	1	0	1	9
EXOC7 (AR)	VT	p.(Ile630Met)/c.1890C>G	F	VUS	22.0	8.55E-05	2	1	2	1	1	1	.	0	0	0	1	9
HSPA1A	GLG/MIT/ER	p.Tyr545*/c.1635C>G	F	VUS	33.0	NF	2	2	3	1	NA	NA	.	0	0	0	1	9
PCM1	MCC	p.(Arg966His)/c.2897G>A	F	VUS	23.5	1.10E-04	2	1	2	1	NA	0	1	0	1	0	1	9
CNGB1 (AR)	CGP	p.Phe856fs/c.2567_2568delTT	M	P	35.0	NF	3	2	3	NA	NA	NA	.	0	0	0	1	9
GLA (XL)	LY/ATP	p.(Asp313Tyr)/c.937G>T	M	B	18.3	4.46E-03	1	1	1	1	1	1	1	0	0	0	1	8
BRAF (AD)	NR	p.(Arg662Ser)/c.1986G>C	F	VUS	21.3	NF	2	1	3	1	0	NA	.	0	0	0	1	8

*: inheritance OMIM; AR: Autosomal Recessive; AD: Autosomal Dominant; XL: X-Linked; LY: Lysosome; ATP: Autophagy; COB: Cobalamin; VT: Vesicular Trafficking; NR: Neuronal; GB: Glycosaminoglycan Biosynthesis; GLG: Golgi apparatus; MIT: Mitochondria; ER: Endoplasmic reticulum; MCC: Mitotic Cell Cycle; CGP: Cyclic Guanosine PKG; INH: Inheritance; M: Mother; F: Father; ACMG: American College of Medical Genetics and Genomics; VUS: Variant of Uncertain Significance; B: Benign; LB: Likely Benign; LP: Likely Pathogenic; P: Pathogenic; CADD: Combined Annotation Dependent Depletion; Freq: Allele frequency; NF: Not Found in gnomAD; MT: MutationTaster; PolP2: PolyPhen-2; SIFT: Sorting Intolerant From Tolerant; F-MKL: FATHM-MKL; GERP: Genomic Evolutionary Rate Profiling; S-AI: SpliceAI; Z/pLI: Z-Loss-of-function; BE: Brain expression; NA: Non applicable; TS: Total score.

- Proteins located at lysosome: LRRK2, GALA (*GLA* gene)
- Lysosomal enzymes: GALA

Supplementary Table 3. Detailed genetic information, pathways, and KDA scores of genetic variants prioritized in patient PD-227.

General Information							Predictor Scores											
**Gene (Inheritance *)**	**Pathway**	**Variant**	**INH**	**ACMG**	**CADD**	**Freq**	**ACMG**	**CADD**	**Freq**	**MT**	**PolP2**	**SIFT**	**F-MKL**	**S-AI**	**GERP**	**Z/pLI**	**BE**	**TS**
KIF16B	VT	p.(Arg613Met)/c.1838G>T	.	VUS	35.0	4.46E-05	2	2	2	1	1	1	1	1	1	0	1	13
ABL2	NR	p.(Arg180Cys)/c.538C>T	.	LP	34.0	8.79E-06	3	2	2	1	1	1	1	0	0	0	1	12
PLD1 (AR)	NR	p.(Arg675Trp)/c.2023C>T	.	VUS	33.0	1.55E-05	2	2	2	1	1	1	1	0	0	0	1	11
UNC13B	NR	p.(Ser263Phe)/c.788C>T	.	VUS	28.8	3.11E-05	2	1	2	1	1	1	1	0	0	0	1	10
LRPPRC (AR)	GLG/MIT/ER	p.(Ile560Leu)/c.1678A>T	.	VUS	26.7	9.54E-04	2	1	2	1	0	1	1	0	1	0	1	10
NPLOC4	GLG/MIT/ER	p.(Asp477His)/c.1429G>C	.	VUS	24.9	1.64E-04	2	1	2	1	0	1	0	0	1	1	1	10
NAPEPLD	NR	p.(Leu378Val)/c.1132T>G	.	VUS	18.4	7.46E-05	2	1	3	1	0	0	1	0	0	0	1	9
ATM (AR,AD)	LY/ATP	p.(Val1729Leu)/c.5185G>C	.	LB	21.3	1.32E-04	1	1	2	1	1	0	1	0	1	0	1	9
EXOC6	VT	p.(Ser639Phe)/c.1916C>T	.	LB	24.1	5.46E-03	1	1	2	1	0	1	1	0	1	0	1	9
GLA (XL)	LY/ATP	p.(Asp313Tyr)/c.937G>T	M	B	18.3	4.46E-03	1	1	1	1	1	1	1	0	0	0	1	8
CNGB1 (AR)	CGP	p.Phe1051fs/c.3150delG	.	P	26.5	7.77E-06	4	1	2	0	0	0	0	0	0	0	1	8
PIK3C2B	IP	p.(Gly8Glu)/c.23G>A	.	LB	21.9	1.48E-03	1	1	1	1	1	0	1	0	1	0	1	8
CHST8	GLG/MIT/ER	p.(Arg307Gly)/c.919C>G	.	VUS	20.4	1.09E-04	2	1	2	0	1	0	1	0	0	0	1	8
HIF1A	LY/ATP/GLG/MIT/ER	p.(Thr442Ile)/c.1325C>T	.	B	22.7	5.97E-03	1	1	1	1	1	0	1	0	1	0	1	8
NOD1	GLG/MIT/ER	p.(Arg637His)/c.1910G>A	.	VUS	21.4	1.93E-03	2	1	1	1	0	0	1	0	1	0	1	8
CLASP1	NR	p.(Met748Thr)/c.2243T>C	.	B	21.8	3.54E-03	1	1	1	1	0	0	1	0	1	1	1	8
BORCS8 (AR)	LY/MAPK	p.(Arg85His)/c.254G>A	.	VUS	22.8	1.35E-05	2	1	2	1	0	0	0	0	1	0	1	8

*: inheritance OMIM; AR: Autosomal Recessive; AD: Autosomal Dominant; XL: X-Linked; VT: Vesicular Trafficking; NR: Neuronal; GLG: Golgi apparatus; MIT: Mitochondria; ER: Endoplasmic reticulum; LY: Lysosome; ATP: Autophagy; MAPK: Mitogen-Activated Protein Kinase; SL: Sphingolipid IP: Inositol Phosphate; CGP: Cyclic Guanosine PKG; INH: Inheritance; M: Mother; ACMG: American College of Medical Genetics and Genomics; VUS: Variant of Uncertain Significance; B: Benign; LB: Likely Benign; LP: Likely Pathogenic; P: Pathogenic; CADD: Combined Annotation Dependent Depletion; FREQ: Allele frequency; MT: MutationTaster; PolP2: PolyPhen-2; SIFT: Sorting Intolerant From Tolerant; F-MKL: FATHM-MKL; GERP: Genomic Evolutionary Rate Profiling; S-AI: SpliceAI; Z/pLI: Z-Loss-of-function; BE: Brain expression; TS: Total score.

- Proteins located at lysosome: BORC8 (*BORCS8* gene), GALA (*GLA* gene)
- Lysosomal enzymes: GALA

Supplementary Table 4. Detailed genetic information, pathways, and KDA scores of genetic variants prioritized in patient PD-216.

General Information							Predictor Scores												
**Gene (Inheritance *)**	**Pathway**	**Variant**	**INH**	**ACMG**	**CADD**	**Freq**	**ACMG**	**CADD**	**Freq**	**MT**	**PolP2**	**SIFT**	**F-MKL**	**S-AI**	**GERP**	**Z/pLI**	**BE**	**TS**
VPS33B (AR)	LY/GLG/ATP	p.(Arg398Cys)/c.1192C>T	F	VUS	33.0	NF	2	2	3	1	1	1	1	0	1	0	1	13
FZD8	NR	p.(Ser410Gly)/c.1228A>G	M	VUS	23.8	3.99E-06	2	1	3	1	1	1	1	0	1	1	1	13
<u>*GLB1* (AR)</u>	LY/ATP/NR	p.(Arg419Gln)/c.1256G>A	F	LP	35.0	7.06E-05	3	2	2	1	0	0	1	1	1	0	1	12
BMP1 (AR)	VT	p.(Asp803Glu)/c.2409C>A	F	VUS	24.1	NF	2	1	3	1	1	1	1	0	1	0	1	12
SPHK2	LY/NR	p.(Gly514Cys)/c.1540G>T	F	VUS	24.6	8.12E-05	2	1	2	1	1	1	1	0	1	0	1	11
SLC4A7	VT	p.(Ile1067Thr)/c.3200T>C	F	VUS	25.9	NF	2	1	3	1	1	0	1	0	1	0	1	11
KIF24	OB	p.(Arg51His)/c.152G>A	M	VUS	29.5	3.56E-05	2	1	2	1	1	1	1	0	1	0	1	11
DAPK1	LY/ATP	p.(Arg666Gln)/c.1997G>A	M	VUS	25.4	6.22E-05	2	1	2	1	0	1	1	0	1	0	1	10
GLTP	VT	p.(Gly114Arg)/c.340G>A	F	VUS	29.5	3.14E-05	2	1	2	1	1	1	0	0	1	0	1	10
STK11 (AD)	LY/ATP	c.*2692G>A	M	VUS	15.4	NF	2	1	3	1	0	0	0	0	1	0	1	9
TSPOAP1 (AR)	NR	p.(Arg9Trp)/c.25C>T	M	VUS	27.6	9.94E-05	2	1	2	0	1	1	0	0	1	0	1	9
MCEE (AR)	GLG/MIT/ER	p.(Arg143His)/c.428G>A	F	B	24.2	2.24E-03	1	1	1	1	1	1	1	0	1	0	1	9
UBQLN1	GLG/MIT/ER/VT	p.(Gly355Val)/c.1064G>T	F	B	26.7	7.77E-03	1	1	1	1	1	1	1	0	1	0	1	9
SEMA3D	AG	p.(Pro615Thr)/c.1843C>A	M	LB	28.4	3.50E-03	1	1	1	1	1	1	1	0	1	0	1	9
ABCA12 (AR)	VT/NR	p.(Val2070Ile)/c.6208G>A	M	B	22.5	1.97E-03	1	1	1	1	1	0	1	0	1	0	1	8
PTCD3 (AR)	GLG/MIT/ER	p.(Arg104His)/c.311G>A	M	LB	17.9	2.81E-04	1	1	2	1	0	0	1	0	1	0	1	8
CLTCL1	VT/NR	p.(Val44Phe)/c.130G>T	F	B	17.9	7.75E-03	1	1	1	1	1	1	1	0	0	0	1	8
LRP2 (AR)	LY/VT	p.(His1099Tyr)/c.3295C>T	M	VUS	16.2	6.97E-05	2	1	2	0	0	0	1	0	1	0	1	8

*: inheritance OMIM; AR: Autosomal Recessive; AD: Autosomal Dominant; LY: Lysosome; ATP: Autophagy; NR: Neuronal; VT: Vesicular Trafficking; OB: Organelle Biogenesis; GLG: Golgi apparatus; MIT: Mitochondria; ER: Endoplasmic reticulum; AG: Axon Guidance; INH: Inheritance; M: Mother; F: Father; ACMG: American College of Medical Genetics and Genomics; VUS: Variant of Uncertain Significance; B: Benign; LB: Likely Benign; LP: Likely Pathogenic; CADD: Combined Annotation Dependent Depletion; Freq: Allele frequency; NF: Not Found in gnomAD; MT: MutationTaster; PolP2: PolyPhen-2; SIFT: Sorting Intolerant From Tolerant; F-MKL: FATHM-MKL; GERP: Genomic Evolutionary Rate Profiling; S-AI: SpliceAI; Z/pLI: Z-Loss-of-function; BE: Brain expression; TS: Total score.

- Proteins located at lysosome: VPS33B, SPHK2 (isoform 2), β-GAL (*GLB1* gene), LRP2.
- Lysosomal enzymes: SPHK2, β-GAL.

Supplementary Table 5. *GLA* variants and GALA activity in parkinsonian patients: reported cohorts and case studies.

Authors/ Patients	Age at onset	Sex	Clinical diagnosis	Parkinsonian clinical features	Clinical testing	GALA activity[1]	*GLA* genotype
Patient series: cohorts of PD patients							
[76]	-	-	PD (N=38)	-	-	Significantly decreased in sporadic PD patients 18.56±1.49 units (leukocytes)	-
[77]	-	M	PD (N=69)	Sporadic PD without family history	-	-	c.-573C>G
[78]	-	-	PD (N=26)	-	-	Low in temporal cortex	-
[18]	-	-	PD (N=648)	-	-	Reduced in PD compared to controls 2.89 nmol/ml/h (leukocytes)	-
[50]	56	F	PD (N=236)	Falls, freezing, wearing off, PoD dyskin, good response to dopaminergic medication	All patients, except one who refused, underwent brain MRI examinations, which revealed only mild subcortical white matter T2 hyperintensities. In one of the subjects, a swallow-tail sign at the level of substantia nigra was revealed. DaTSCAN in 4 patients	-	**p.Asp313Tyr**
	61	F		Tremor, PoD dyskinesia, wearing off, good response to dopaminergic medication		-	**p.Asp313Tyr**
	69	F		Freezing, PoD dyskinesia, good response to dopaminergic medication		-	**p.Asp313Tyr**
	60	F		Freezing, tremor, PoD dyskinesia, wearing off, good response to dopaminergic medication		-	**p.Asp313Tyr**
[20]	65	M	PD (N=277)	Tremor-dominant subtype. Non-motor symptoms: cognitive impairment. Good response to levodopa	-	2.2 nmol/ml/h (dry blood spot)	**p.Asp313Tyr**
	63	F		Tremor-dominant subtype. Non-motor symptoms: urinary dysfunction, constipation and fatigue. Good response to levodopa	Deep brain stimulation	2.6 nmol/ml/h (dry blood spot)	**p.Asp313Tyr**

	42	F		Tremor-dominant subtype. Non-motor symptoms: urinary disfunction. Good response to levodopa	-	2.8 nmol/ml/h (dry blood spot)	**p.Asp313Tyr**
	27	F		Postural instability and gait disorder. Non-motor symptoms: urinary dysfunction and fatigue. Good response to levodopa	MRI: bifrontal and periventricular ischemic gliotic areas. Cerebellar atrophy and right temporal encephalomalacia	3.5 nmol/ml/h (dry blood spot)	p.Leu347Ser
[79]	-	-	PD (N=144)	Tremor-dominant subtype (53%), akinetic-rigid (28%), mixed (15%), postural instability and gait disorder (4%). Good response to dopaminergic treatment in all patients	-	20.7±5.6 nmol/ml/h (leukocytes)	No relevant *GLA* variants detected
[19]	-	-	PD (N=66)	-	-	Significantly lower α-gal activity 1.75±0.34 nmol/ml/h (serum)	-
[14]	69	M	PD (N=252)	Hypokinetic-rigid type, right side emphasized	MRI: falxmeningeoma, moderate cerebral microangiopathy and global cerebral atrophy. DaTSCAN positive	2.4 nmol/ml/h	**p.Asp313Tyr**
	56	F		Mixed-type, right side emphasized	MRI: moderate internal cerebral atrophy, isolated periventricular microangiopathic lesions. DaTSCAN positive	2.5 nmol/ml/h	**p.Asp313Tyr**
	62	M		Hypokinetic-rigid type, right side emphasized	MRI: moderate cerebral microangiopathy, with beginning status lacunaris of basal ganglia, global cerebral atrophy emphasized temporal on the right side without temporo mesial atrophy	2.1 nmol/ml/h	**p.Asp313Tyr**
	60	F		Hypokinetic-rigid type, left side emphasized	Lowgrade cerebral microangiopathy	4.6 nmol/ml/h	p.Asp182Asn

[1]GALA activity determined in different tissues (depending on the study) (normal ranges: 15-56 nmol/mg/h consulted in Vall d'Hebrón Hospital, Barcelona; 11.3-38.7 nmol/ml/h [79].

F, Female; FD, Fabry Disease; M, Male; MRI, Magnetic Resonance Imaging; PD, Parkinson's Disease. PoD, peak-of-dose

Supplementary Table 6. *In silico* predicted effects of the *GLA* (p.Asp313Tyr) and *GLB1* (p.Arg419Gln) variants found in the patients PD-302, PD-227, and PD-216.

Gene (RefSeq)[1]	Variant	MAF[2]	CSVS[3]	CADD[4]	REVEL[5]	GERP[6]
GLA (NM_000169.2)	c.937G>T p.(Asp313Tyr)	0.0037	NR	16.12	0.61	5.46
GLB1 (NM_000404.4)	c.1256G>A p.(Arg419Gln)	0.00004	NR	35	0.52	5.46

[1]Reference Sequence
[2]gnomAD (v 4.01). Genome Aggregation Database (European non-finish frequency). http://gnomad.broadinstitute.org
[3]CSVS. Collaborative Spanish Variant Server. http://csvs.babelomics.org
[4]CADD. Combined Annotation Dependent Depletion. The variant is pathogenic above 15. https://cadd.gs.washington.edu
[5]REVEL. Rare Exome Variant Ensemble Learner. Scores range from 0 (benign) to 1 (pathogenic).
[6]GERP. Genomic Evolutionary Rate Profiling. Scores ranges from -12.3 (low conserved) to 6.17 (high conserved).
NR: not reported
